# TreeShrink: fast and accurate detection of outlier long branches in collections of phylogenetic trees

**DOI:** 10.1186/s12864-018-4620-2

**Published:** 2018-05-08

**Authors:** Uyen Mai, Siavash Mirarab

**Affiliations:** 1 Computer Science and Engineering, University of California at San Diego, San Diego, 92093 CA USA; 2 Electrical and Computer Engineering, University of California at San Diego, San Diego, 92093 CA USA

**Keywords:** Tree diameter, Rogue taxon removal, Gene tree discordance

## Abstract

**Background:**

Sequence data used in reconstructing phylogenetic trees may include various sources of error. Typically errors are detected at the sequence level, but when missed, the erroneous sequences often appear as unexpectedly long branches in the inferred phylogeny.

**Results:**

We propose an automatic method to detect such errors. We build a phylogeny including all the data then detect sequences that artificially inflate the tree diameter. We formulate an optimization problem, called the *k*-shrink problem, that seeks to find *k* leaves that could be removed to maximally reduce the tree diameter. We present an algorithm to find the exact solution for this problem in polynomial time. We then use several statistical tests to find outlier species that have an unexpectedly high impact on the tree diameter. These tests can use a single tree or a set of related gene trees and can also adjust to species-specific patterns of branch length. The resulting method is called TreeShrink. We test our method on six phylogenomic biological datasets and an HIV dataset and show that the method successfully detects and removes long branches. TreeShrink removes sequences more conservatively than rogue taxon removal and often reduces gene tree discordance more than rogue taxon removal once the amount of filtering is controlled.

**Conclusions:**

TreeShrink is an effective method for detecting sequences that lead to unrealistically long branch lengths in phylogenetic trees. The tool is publicly available at https://github.com/uym2/TreeShrink.

**Electronic supplementary material:**

The online version of this article (10.1186/s12864-018-4620-2) contains supplementary material, which is available to authorized users.

## Background

Datasets used in phylogenetic analyses include a large number of genes and species these days. The number of loci involved and the size of the trees make it impossible to carefully examine every sequence alignment and every gene tree manually. Such manual curation, even if possible, is subject to biases of the curator and poses challenges in reproducibility. But the need for data curation is as strong as ever. Phylogenetic analyses typically use pipelines of many steps, starting from sequencing, to contamination removal, homology and orthology detection, multiple sequence alignment, and gene tree inference, and finally species tree reconstruction. Each step is error-prone, and it has been long recognized that errors can propagate among these steps [1–3]. However, detecting errors is difficult, especially when large numbers of genes are being analyzed [4]. For example, discordance among estimated gene trees may have biological causes or may be the result of gene tree estimation error; when error-prone gene trees are fed to a species tree estimation method, the error may propagate [5–7]. This possibility motivates the co-estimation methods that aim to break or weaken the chain of error propagation [8–10]. However, the end-to-end co-estimation of all steps in the phylogenetic analyses remains elusive [10]. In practice, analysts often devise creative (if ad-hoc) methods to find and remove erroneous data. Such data filtering should be treated with care because it may remove useful signal in addition to error [11], and it also runs the risk of introducing biases. One common method of data filtering is alignment masking [12, 13], despite some criticism [11]. Beyond filtering based on sequences, detecting problematic species from reconstructed trees is also possible.

Two common approaches for filtering based on phylogenetic trees are rogue taxon removal (RTR) [14–18] and gene tree filtering [19, 20]. More recent approaches include filtering of individual sites with an outsized impact on the tree topology [21]. RTR aim to find species that have an unstable position in the inferred trees, judging the stability with regards to replicate trees generated by bootstrapping [16, 18] or jackknifing [14]. A second approach is to remove genes that are believed to be problematic, perhaps due to missing data [19, 20], lack of signal [22], or even inconsistent signal [21]. When potentially problematic genes are removed, the justification is that the inference of the species tree (i.e., by summarizing gene trees or concatenation) may become more accurate as a result. Alternatively, some analyses (e.g., [23, 24]) filter individual species from individual gene trees based on some criteria (e.g., fragmentation) while keeping the gene. These analyses aim to find and eliminate only the problematic data but nothing more.

**Fig. 1:**
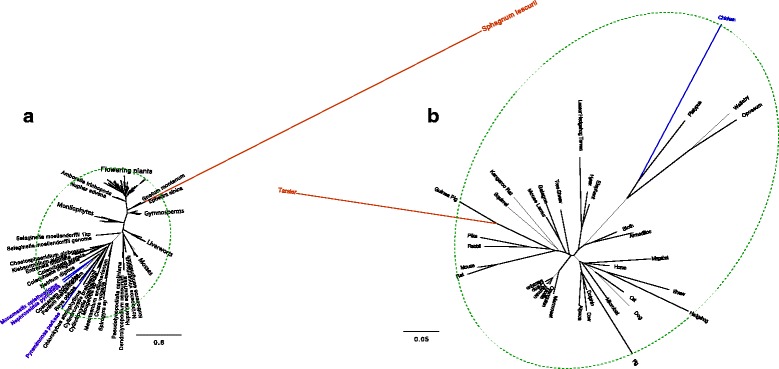
Example trees with suspicious long branches. **a** An unfiltered gene tree of a Plant dataset [23] with an obvious outlier leaf; **b** a gene tree in a mammalian dataset with a hard to detect outlier branch [36]. Outgroups are shown in blue and the suspicious long branches in the red. Dashed green line: the tree diameter after removal of red branches. Detecting the red branch is easy on the left but hard on the right

The branch lengths of an inferred phylogeny can provide indications of error in sequence data in some cases. If the evolution follows a strict molecular clock, we expect that all leaves should be equidistant from the root. Deviations from the strict clock, if not extreme, would not produce situations where a small minority of species have *dramatically* different rates of evolution and hence root to tip distances. In other words, variations in root to tip distance are expected, but outlier species in terms of distances to the root have to be treated with suspicion. Several types of error in a phylogenetic data, e.g., contamination, mistaken orthology, and misalignment, can lead to the addition of very long branches to the tree (e.g., Fig. 1a). When a handful of species dramatically diverge from the rest, it is likely that the sequences of outlier species contain errors (of unclear nature). Also suspicious is a species that has normal root to tip distances in most gene trees but has an unexpectedly large root to tip distance in a handful of genes. Even when the sequences of long branches are error-free, they may still pose difficulties due to long branch attraction [25]. Thus, several studies have tried removing species with outlier root-to-tip distances in gene trees [23, 26]. However, rooting is often challenging and prone to error [27]. Moreover, rooting is not necessary for finding outlier species in terms of branch length.

A useful concept is the tree diameter, which gives the maximum distance between any two leaves of the tree. We introduce an optimization problem that if solved efficiently can help in finding species that artificially inflate the tree diameter. The *k*-shrink problem: Given a tree on *n* leaves with branch lengths and a number 1≤*k*≤*n*; for every 1≤*i*≤*k*, find a set of *i* leaves that should be removed to reduce the tree diameter maximally.

We develop an algorithm to solve the problem in $O\left (k^{2}h+n\right)$ time where *h* is the height of the tree after being rerooted at the centroid edge (which can be done in linear time [27]). Given the solution to the *k*-shrink problem, we need to decide the species to remove such that the number of error-free sequences removed is minimized. Towards this goal, we propose three statistical tests to find outlier species. We set $k=\Theta (\sqrt {n})$ and compute the proportional reduction in the diameter when going from *i*−1 to *i* removals for 1≤*i*≤*k*. We then look for outlier values in the spectrum of these proportional reductions; outliers are defined as those that lie at the extreme tails of the distribution, and the outlier detection is controlled by a level of false positive tolerance (*α*). A further complication is that outgroups, even when error-free, can greatly impact the diameter (Fig. 1b). Moreover, if a clade has an increased rate of mutations, it may impact the tree diameter more than other clades and may become prone to removal. When multiple gene trees are available, we can learn such patterns of rate variation. Our second statistical test simply combines data from all gene trees to find outliers in a single distribution. The third test goes further and learns a different distribution per species. We implement these tests in a tool called TreeShrink.

We test TreeShrink on six phylogenomic datasets and an HIV transmission tree. We show that TreeShrink improves the quality of gene trees effectively for phylogenomic datasets and can separate strains of HIV. When distributions are learned per species, outgroups are also handled effectively.

## Methods

### Notations and definitions

For an unrooted tree *t* on the leaf-set $\mathcal {L}$, let *δ*(*a*,*b*) give the distance between *a* and *b*. The restriction of *t* to the leafset *A* is denoted by $t\restriction _{A}$, and we use the shorthand $t\backslash _{a} = t\restriction _{\mathcal {L} t - \{a\}}$. We refer to a pair of leaves in *t* with the highest pairwise distance as a *diameter pair* and call the two leaves *on-diameter*. Any tree has at least one diameter-pair but could have more. We define $\mathcal {P}(t)$ as a set of all diameter pairs of *t*; that is, $ \mathcal {P}(t)=\{(a,b) : (\forall x,y\in \mathcal {L} t) [{\delta } (a, b) \geq {\delta } (x, y)]\} $. The diameter set $\mathcal {D}(t)$ is defined as the set of all on-diameter leaves: $ \mathcal {D}(t)=\{a : (\exists x)[(a,x)\in \mathcal {P}(t)] \} $.

We call a tree *tsingly paired* if *all* the restricted trees of *t* (including *t*) have only one diameter pair; that is, $\forall A \subseteq \mathcal {L} t, |\mathcal {P}(t\restriction _{A})|=1$. We refer to the process of removing one leaf from *t* as a *removal*. A removal is called *reasonable* iff $a \in \mathcal {D}(t)$.

A *removing chain* of *t* is defined as an ordered list of removals. We refer to a removing chain of length *k* as a *k-removing chain* and denote it by $\mathcal {H}_{k}(t)$. We refer to a removing chain that consists only of reasonable removals as a *reasonable removing chain*. An *optimal k-removing chain*, $\mathcal {H}^{*}_{k}(t)$, is a removing chain that results in a tree with the minimum diameter among all chains of length *k*. Any $\mathcal {R}_{k}(t)\subset \mathcal {L} t$ with $|\mathcal {R}_{k}(t)|=k$ is called a *k-removing set* of *t*, and is called a *reasonable k-removing set* if there exists an ordering of $\mathcal {R}_{k}(t)$ that gives a reasonable *k*-removing chain. We refer to the set of all reasonable *k*-removing sets as the *k-removing space* of *t*, and denote it by $\mathcal {S}_{k}(t)$. We let $\mathcal {R}^{*}_{k}(t)$ denote an arbitrary *removing set* that gives the restricted tree with the minimum diameter. Finally, for a rooted version of *t*, we let *Cld*(*u*) denote the set of leaves descended from *u*.

For all the theoretical results given below, proofs are given in the Additional file 1: Appendix.

### A polynomial time solution to the *k*-shrink problem

Only reasonable removals have the potential to reduce the tree diameter. If *t* is singly paired, two reasonable removals exist, and one of them *may* reduce the diameter more. This can be simply checked and thus, the problem is trivial for *k*=1. For *k*>1, a greedy approach that takes the optimal removal at each step does not always produce an optimal solution (see Additional file 1: Figure S1a for a counter-example). Therefore, to solve this problem, we need to consider a search space. However, a brute force search for all reasonable k-removing chains is infeasible. The brute force method would first consider the initial diameter pair(s); then, to remove each of the two on-diameter leaves, it would consider the new diameter pair(s) after the first removal and recurse on each diameter-pair. This recursive process produces all reasonable removing chains from 1 to *k*, but its space grows exponentially.

Three observations enable us to find the optimal solution in a reduced search space that only grows linearly with *k*. The first observation is that if (*a*,*b*) is a diameter pair, then *b* remains on-diameter after removing *a*.

#### **Proposition 1**

If an on-diameter leaf is removed, the rest of the on-diameter set are on-diameter for the restricted tree: $a\in \mathcal {D}(t) \Rightarrow \mathcal {D}(t) - \{a\} \subset \mathcal {D}(t\backslash _{a})$.

**Fig. 2:**
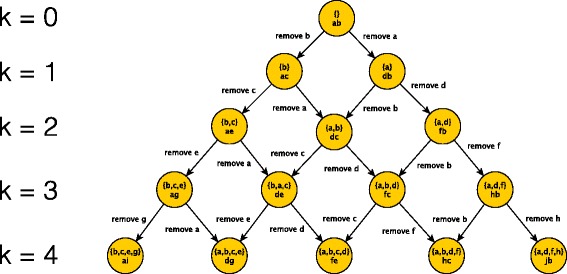
Graphical representation of the reasonable search space. The root node represents the initial tree *t*; each node on row *k* represents a restricted tree with *k* leaves removed. Each node is annotated by the removing set (top) and a diameter pair of the induced tree (bottom). Each edge in the graph represents a reasonable removal. The path from the root to any node corresponds to a reasonable removing chain. Each row *k* in the graph gives the *k*-removing space of *t* ($\mathcal {S}_{k}(t)$)

All *i*-removing spaces for 1≤*i*≤*k* can be represented as a directed acyclic graph (Fig. 2). In this DAG, each node at row *i* represents an *i*-removing set $\mathcal {R}_{i}(t)$, and is also annotated with a diameter pair after the removal of $\mathcal {R}_{i}(t)$. All the entries in the row *i* form the *i*-removing space. Any path from the root ending at a node $\mathcal {R}_{i}(t)$ is an *i*-removing chain. Note that each node can be reached with multiple paths from the root; this leads to a second observation, which is trivial but important. Any ordering of an *i*-removing chain gives the same restricted tree. Thus, we can reduce the search space from reasonable chains to reasonable sets. The first two observations allow us to design a polynomial time algorithm for singly paired trees (described next). Our third observation (formalized later) is that when a tree is not singly paired, breaking ties arbitrarily still guarantees optimality.

#### Singly paired trees

Our main result states that, for singly paired trees, the *i*^*th*^ row of the reasonable search space graph (Fig. 2) contains *i*+1 nodes and one of the nodes gives an optimal *i*-removing set. Moreover, traversing all *O*(*k*^2^) nodes in this graph gives the optimal solution to our *k*-shrink problem.

##### **Theorem 1**

The *k*-removing space of a singly paired tree *t* includes all the optimal *k*-removing sets of *t*; that is: $\forall k>0: \mathcal {R}^{*}_{k}(t) \in \mathcal {S}_{k}(t)$.

##### **Theorem 2**

The size of the *k*-removing space for a singly paired tree *t* is *k*+1.

##### **Corollary 1**

The size of the reasonable search space up to level *k* is *O*(*k*^2^).



Our algorithm (Algorithm 1) start with a preprocessing in order to enable computing the pair set at any point in a removal chain in *O*(*n*). The preprocessing uses a bottom-up traversal of *t* (rooted arbitrarily). For each internal node *u*, we store four values: (i) the leaf *x*∈*Cld*(*u*) with the longest distance to *u*, (ii) the distance *δ*(*u*,*x*), (iii) the leaf *y*∈*Cld*(*u*)−*Cld*(*c*_1_) with the longest distance to *u* (where *c*_1_ is a child of *u* such that *x*∈*Cld*(*c*_1_)), and (iv) the distance *δ*(*x*,*y*) (see Additional file 1: Figure S1b). We store these values for each node *u* as a tuple *rec*(*u*)=(*rec*_1_(*u*),*rec*_2_(*u*),*rec*_3_(*u*),*rec*_4_(*u*)). These values can be computed in a post-order traversal of the tree in the natural way. Once these records are computed, finding diameter pairs can be done quickly (see function FindPair in Algorithm 1). Let (*a*,*b*) be a diameter pair; note that regardless of the arbitrary rooting of the tree, at the LCA of *a* and *b*, the record includes *a*, *b* as the first and third fields and the tree diameter as the last. Thus, the tree diameter corresponds to the record with the largest fourth value. As we will see, throughout the algorithm, the values of the records may have to change. However, these updates can also happen in *O*(*h*). Thus,

##### **Proposition 2**

Given a rooted tree *t* of height *h*, $(a,b) \in \mathcal {P}(t)$, and *rec*(*u*)*u* for all nodes $u \in \mathcal {L} t$, we can find one diameter pair of *t*∖_*a*_ in *O*(*h*).

Once the preprocessing finishes, we start building the DAG (Fig. 2). We start with the root node, corresponding to the initial tree *t* and build the rows iteratively. For any node at step *i* with (*x*,*y*) as its diameter pair, two nodes have to be added to the next row, one for removing *x* and another for removing *y*. As the proof of Theorem 2 (Additional file 1: Appendix) indicates, two sister nodes in step *i* have to share one descendant in step *i*+1 (Fig. 2). Thus, to construct each row from the previous row we simply need to find the diameter pair of the tree restricted to the removal-set of each node; this is done in the function FindPair previously described. As we build the DAG, we also keep track of the length of the diameter at each node and the optimal *i*-removing set. At the end, we report an optimal-removing set for each *i* from 1 to *k*.

According to Proposition 2, finding each new diameter pair after removing any node can be done in *O*(*h*). From Corollary 1 and Proposition 2, we have:

##### **Corollary 2**

Algorithm *1* solves the *k*-shrink problem in $O\left (k^{2}h+n\right)$.

#### Generalization to all trees

If the tree *t* is not singly paired, nodes in the search graph could have more than two children which increase the size of the search space. However, we prove that we can break the ties arbitrarily at any step and still guarantee *an* optimal solution. It follows naturally that Algorithm 1 also works for trees that are not singly paired.

For any diameter pair $(a,b)\in \mathcal {P}(t)$, we define a *pair-restricted k-removing space* as a subset of $\mathcal {S}_{k}(t)$ such that each of its elements includes either *a* or *b*.

##### **Theorem 3**

For any *k*, any arbitrary pair-restricted *k*-removing space includes at least one optimal *k*-removing set.

##### *Proof*

(sketch). It is not hard to prove that any tree *t* has a single midpoint which partitions its diameter set into disjoint subsets. We call each of those subsets a *diameter group* of *t* (Additional file 1, Appendix A, Lemma S2 and Lemma S3). Clearly, unless all but one of the diameter groups are removed, the tree diameter is unchanged. We refer to the restricted tree of *t* that have all but one of the diameter groups removed as a *minimum shrunk tree* of *t*. We can prove that any arbitrary pair-restricted removing space can produce *all* minimum shrunk tree (see the full proof in the Additional file 1: Appendix). If *k* is so small such that there is no *k*-removing set can reduce the tree diameter, any solution is optimal and the result of Theorem 3 trivially follows. Otherwise, any optimal solution of *k*-shrink can be induced from one of the minimum shrunk trees (Lemma S4 in Appendix A, Additional file 1). Thus, to find an optimal tree *t*^∗^, we can start from *any* pair-restricted removing space and concatenate the two removing chains: the chain that induces the minimum shrunk tree $t_{i}^{*}$ from any arbitrary diameter pair, and the chain that starts from $t_{i}^{*}$ to induce *t*^∗^. Full proof is given in Appendix A of Additional file 1. □

According to Theorem 3, any pair-restricted *k*-removing space includes at least one optimal solution. For a tree that is not singly paired, we can arbitrarily restrict the search space to any of its diameter pairs *at any step* of the algorithm. This ensures that the search space size grows with *O*(*k*^2^), and that Algorithm 1 still correctly finds an optimal solution in $O\left (k^{2}h+n\right)$.

### Statistical selection of the filtering species

The solution to the *k*-shrink problem for a given *k* gives the minimum diameters for 1≤*i*≤*k* and the corresponding optimal removing sets. Given these results, we now need to find a set of species that have *unexpectedly* large impacts on the tree diameter. Defining what is an expected impact on the diameter is not trivial and depends on many factors such as the rate of speciation, taxon sampling, and the tree topology. To avoid modeling such processes, we use empirical statistics.

**Fig. 3:**
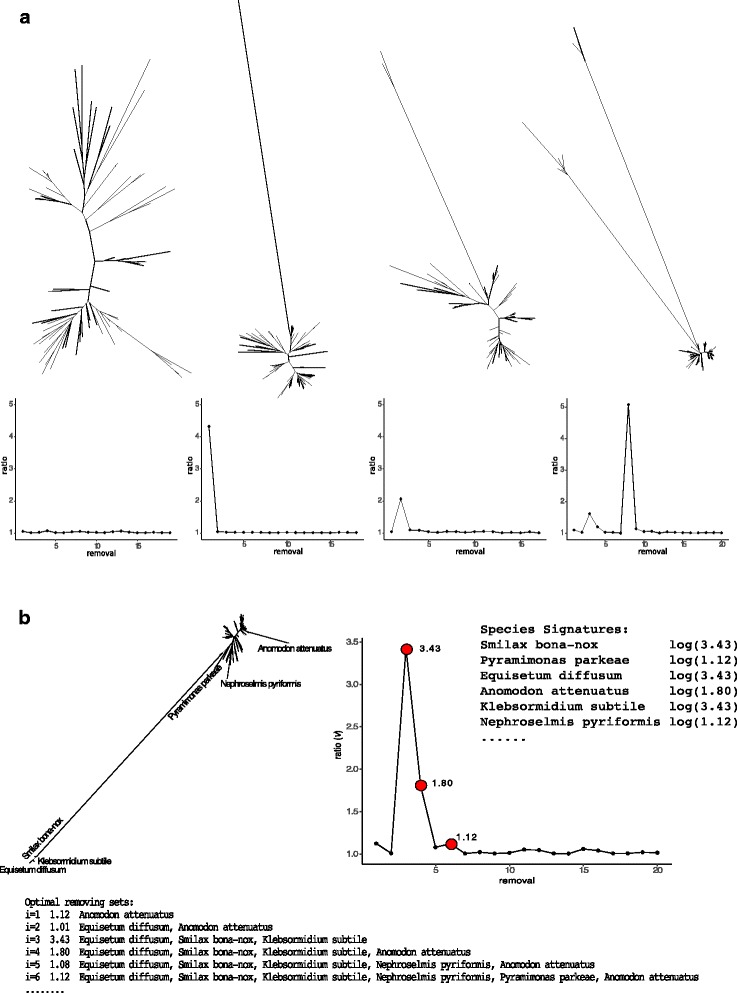
**a** Patterns of *ν*_*i*_ as a function of *i*. Four unfiltered gene trees from a Plant dataset [23] are shown (*top*). For each tree, we also show *ν*_*i*_ for $1\leq i\leq k=min(n/4,5\sqrt {n})$ (*bottom*). **b** An example tree from the Plant dataset with the removing sets and species signatures. The removing sets are shown with the corresponding *ν* values. The max *ν* values associated with the species signatures are marked in red

Let *ν*_*i*_ be the ratio of the minimum diameter with *i*−1 leaves removed and the minimum diameter with *i* leaves removed, and let ${\Delta }_{i}=\log ({\nu }_{i})$. For a tree with no outlier branches, we expect *ν*_*i*_ values to be close to one (e.g., T1 in Fig. 3a). For a tree with one outlier leaf on a very long branch, we expect that *ν*_1_ is much larger than other *ν*_*i*_ values (T2 in Fig. 3a). If two species are on a very long branch, we expect a small *ν*_1_, a large *ν*_2_, and small values again for *i*>2 (T3 in Fig. 3a). If there are two exceptionally long branches, one with three species and another with five species, we expect *ν*_3_ and *ν*_8_ to be large and other values to be small (T4 in Fig. 3a). We use *ν* values to detect outliers, but we first need to introduce the concept of a *signature*.

The *ν*_*i*_ values for the removing sets that include a species measure the impact of that species on the tree diameter. We will refer to the maximum *Δ*_*i*_ among all removing sets *i* that include a species as the *signature* of that species (note that this is defined only for some of the species). A species with an exceptionally larger signature compared to the other species can be considered an outlier (Fig. 3b). To define what qualifies as exceptionally large, we design three different tests. The first test can be applied to a single tree, while the other two require a collection of gene trees.

#### The “per-gene” test

For a single input tree and a large enough *k*, we have a distribution over signature values. Since we have limited data in this scenario, we use a parametric approach and fit a log-normal distribution to the signatures. Given a false positive tolerance rate *α*, we define values with a CDF above 1−*α* as outliers. Then, species associated with the outlier signatures are removed.

#### The “all-gene” test

When a dataset includes several gene trees, all related by a species tree, combining the distributions across genes can increase the power. With many genes, we may also be able to distinguish outgroup species from outliers. The signatures of outgroups across all gene trees should be consistently higher than those of ingroups, and these high signatures will appear as part of the combined signature distribution. Thus, we may be able to avoid designating outgroups signatures as outliers.

In this test, we put the signature of all genes together to create one distribution. Unlike the per-gene test, here we have many data points, which enables us to use a non-parametric approach. We compute a kernel density function [28] over the empirical distribution of the combined set of signature values. To estimate the density, we use Gaussian kernels with Silverman’s rule of thumb smoothing bandwidth [28] (as implemented in the R package [29]). Given the density function and a false positive tolerance rate *α*, we define values with a CDF above 1−*α* as outliers.

#### The “per-species” test

Outgroups can contribute to the tree diameter as much as erroneous species (Fig. 1b). To better distinguish outgroups from errors, when a set of gene trees are available, we can learn a distribution *per* species. Given a sufficient number of gene trees, the signatures of a species across all genes form a distribution that specifically captures the impact of that species on the gene tree diameter. These species-specific distributions naturally model the inherent difference between outgroups and ingroups in terms of their impacts on the tree diameter. More broadly, changes in the evolutionary tempo are captured naturally by the per species distributions.

In this test, we first compute a non-parametric distribution of the signature values for each species. When the signature of a species is not defined for a gene, we simply use zero as its signature. Then, for each species, we use the same non-parametric approach as in the all-gene test to compute a threshold for the signature value corresponding to the chosen *α*. Finally, we remove each species from those genes where its signature is strictly above its species-specific threshold.

#### The default parameters of TreeShrink

TreeShrink has two parameters: *α* and *k*. By default, we set *α* to 0.05 (but users can choose other thresholds). Large values of *k* do not fit our goal of finding *outlier* species and can even lead to misleading results (e.g., Figure S2 in Appendix B, Additional file 1), but a small value of *k* may also miss outliers and may lead to insufficient data points for learning distributions.

Using a value of *k* that grows sublinearly with *n* (i.e., the number of leaves) gives us an algorithm that is fast enough for large *n*. For example, using $k=\Theta (\sqrt {n})$ gives *O*(*nh*) running time, which on average is close to $O(n\log n)$ and is *O*(*n*^2^) in the worst case. While the choice must be ultimately made by the user, as a default, we set $k=min\left (\frac {n}{4},5\sqrt {n}\right)$. This heuristic formula ensures that our running time does not grow worse than quadratically with *n* but also avoids setting *k* to values close to *n* (thus also limits the proportion of leaves that *could* be removed).

### Evaluation procedures

#### Datasets

We use six multi-gene datasets and a single-gene HIV dataset, and each dataset includes one or more outgroup species defined by the original papers (Table 1).

**Table 1:** Summary of the biological datasets

Dataset	Species	Genes	Outgroups	Download
Plants [23]	104	852	Monomastix opisthostigma,	DOI 10.1186/2047-217X-3-17
			Uronema sp.,	
			Nephroselmis pyriformis,	
			Pyramimonas parkeae	
Mammals [36]	37	424	Chicken	DOI 10.13012/C5BG2KWG
Insects [31]	144	1478	IXODES SCAPULARIS,	http://esayyari.github.io/InsectsData
			Symphylella vulgaris,	
			Glomeris pustulata,	
			Lepeophtheirus salmonis,	
			DAPHNIA PULEX,	
			Cypridininae sp,	
			Sarsinebalia urgorii,	
			Celuca puligator,	
			Litopenaeus vannamei	
Cannon [32]	78	213	Salpingoeca rosetta,	DOI 10.5061/dryad.493b7
			Monosiga brevicollis	
			Mnemiopsis leidyi,	
			Pleurobrachia bachei,	
			Euplokamis dunlapae	
Rouse [33]	26	393	Mnemiopsis leidyi,	DOI 10.5061/dryad.79dq1
			Amphimedon queenslandica,	
			Trichoplax adhaerens,	
			Nematostella vectensis	
Frogs [39]	164	95	Latimeria chalumnae,	DOI 10.5061/dryad.12546.2
			Protopterus annectens,	
			Homo sapiens,	
			Crocodylus siamensis,	
			Gallus gallus,	
			Ichthyophis bannanicus,	
			Batrachuperus yenyuanensis,	
			Andrias davidianus	

##### Plants [23]:

This dataset of 104 plant/algae species (four Chlorophyta outgroups) and 852 genes was used to establish early diversification patterns within land plants and their sister groups. The data are based on transcriptoms, and authors faced challenges in terms of gene identification and annotation, leading to abundant missing data. To obtain reliable species trees using ASTRAL [30], the authors had to use various filterings, including removal of low occupancy genes and genes with fragmentary sequences. The ASTRAL tree obtained on these filtered gene trees was mostly congruent with results from concatenation, though some interesting clades (e.g., the Bryophytes) were differently resolved. In our analyses, we start with all gene trees estimated from nucleotide data with the third codon position removed.

##### Insects [31]:

This phylotranscriptomic dataset includes 144 species and 1478 genes. This dataset was used to resolve controversial relationships among major insect orders, but only concatenation analyses were reported. A different paper performed a species tree analysis of the same dataset using ASTRAL, obtained on RAxML gene trees that we estimated on all 1478 gene trees [24]. We use these gene trees in our analysis.

##### Metazoa-Cannon [32] and Rouse [33]:

Whether Xenacoelomorpha (a group of bilaterally symmetrical marine worms) are sister to all the remaining Bilateria (animals with bilateral symmetry) has been the subject of much recent debate [32–34]. The Cannon et al. dataset of 213 genes from 78 species sampled from across the animal tree-of-life was used to confidently place Xenacoelomorpha as sister to Bilateria. Among other analyses, ASTRAL-II [35] was used on a collection of gene trees that the authors published, and we use. The dataset by Rouse et al. addresses the same question as Cannon et al. using 393 genes and 26 species.

##### Mammals [36]:

This mammalian dataset consists of 37 species (36 mammals and Chicken as outgroup) and 424 gene trees. Since the original gene trees had several issues (including insufficient ML searches and mislabeled species [37]), here we use RAxML gene trees that were inferred and used in a re-analysis of this dataset [6]. Several reanalyses of this dataset using various methodologies have largely been consistent, except, for the position of the tree shrews that often changes [6, 30].

##### Frogs [38]:

This dataset consists of 164 species (156 frog species and 8 outgroups) and 95 genes. The dataset was used to study the evolutionary history and tempo of frog diversification [38]. The RAxML gene trees we use here were used as inputs for ASTRAL to construct the species tree [38] and were provided by the authors [39].

##### HIV dataset [40]:

This HIV dataset consists of 648 partial HIV-1 *pol* sequences that were used to reconstruct the local HIV-1 transmission network from 1996 to 2011 in San Diego, California. The dataset consists of 639 subtype B, 7 non-subtype B, and 2 unassigned sequences of HIV-1 *pol* coding region. The sequences have GenBank accession numbers from KJ722809 to KJ723456, and were provided to us by the authors. Note that this dataset has only one gene.

#### Methods tested

We implemented TreeShrink (https://github.com/uym2/TreeShrink) using the Dendropy package [41]. We compare the three tests of TreeShrink, namely per-gene, all-gene, and per-species. In addition, we compare the most effective test of TreeShrink, the per-species test, with two alternative methods and a control where we remove species randomly from the tree.

The main alternative to TreeShrink used previously [23, 26] is to root gene trees and then remove species with outlier root-to-tip distances. We use this “rooted pruning” approach where we define outliers as values that lie several standard deviations (we vary this threshold) above the average. For the Plant dataset, 681 genes included the outgroups; for the remaining, we used a linear-time implementation of the midpoint rooting [27]. In other datasets, each gene tree included at least one of the outgroups. While the goals of RTR are somewhat different from ours, we also compare our method with RogueNaRok [16], which defines a rogue taxon as one that has unstable positions in replicate bootstrap runs.

#### Evaluation

Judging the effectiveness of the filtering methods on real data is challenging, as we do not know if a removed sequence is in fact erroneous. However, patterns of discordance can help. While true gene trees may be discordant with the species tree, erroneous sequences will further increase the observed discordance. Thus, the amount of gene tree discordance among genes should reduce as a result of effective filtering, and more effective filtering methods arguably reduce the discordance more than less effective ones. Thus, the quality of a filtering procedure can be judged (albeit with some uncertainty) by its impact on gene tree discordance, as long as its optimization problem does not seek to reduce discordance directly. Note that none of the methods that we test take the species tree as input, and none is trying to directly reduce the gene tree discordance. Thus, we use the reduction in discordance as one measure of accuracy. To compute gene tree discordance, we compare all pairs of gene trees to each other and use the MS (Matching Splits) metric [42], implemented in the TreeCmp [43] to measure distance. To facilitate the interpretation of MS, which is not normalized, we include random removal as a control.

A second concern is the potential of methods to aggressively remove true signal. To evaluate this, we investigate the impact of filtering on the taxon occupancy, defined as the number of gene trees that include each species. Lowered occupancy may negatively impact downstream analyses such as species tree inference and functional analyses. Ideally, a filtering method would not reduce taxon occupancy dramatically. Moreover, removing the same species repeatedly from many genes could be even more problematic for downstream analyses such as species tree estimation.

Filtering methods have a knob that can control the amount of filtering. To avoid impacts of arbitrary choices, we explore a range of knob settings. For the three tests of TreeShrink, we set *α* to 20 different values in the range $\left [0.005,0.1\right ]$. For RogueNaRok, we change the weight factor to control the penalizing factor of the dropset size by setting it to 21 values in range $\left [0,1.0\right ]$ (0.0 is the default value). For rooted pruning, we vary the number of standard deviations above the average that would constitute long branches between 0.25 to 5.00, with 0.25 increments. For random pruning, for each threshold of TreeShrink on each gene tree, we remove the same exact number of leaves as TreeShrink removes, but we choose the species randomly. We repeat the random pruning ten times and show the average.

On the HIV dataset, we test the power of TreeShrink (*α*=5*%*), rooted pruning (3 std), and RogueNaRok (default settings) in detecting the outliers. Outliers are either non-subtype B sequences in the full dataset in experiment 1 or the simulated outliers we added in experiment 2 (described below).

In the first experiment, we infer a RAxML tree from the 648 sequences and use it as the input for TreeShrink. We root the RAxML tree at its midpoint and use it for rooted pruning. To run RogueNaRok, we also create 100 bootstrap trees using RAxML. We use the 7 non-subtype B and 2 unassigned sequences as *outliers* (see Additional file 1, Appendix B, Table S1) and test if TreeShrink, rooted pruning, and RogueNaRok can detect them.

In the second experiment, we add 10 simulated outliers to the 639 subtype B sequences and use TreeShrink and rooted pruning to detect them. To create the outliers, we randomly select 10 sequences from the 639 subtype B sequences and change a small fraction of their sites, selected randomly, to a random nucleotide drawn from the distribution of the base frequencies estimated from the original sequences. In order to root the tree, we include the 3 subtype C sequences (Table S1 in Appendix B, Additional file 1) and root the tree on the branch separating the two subtypes, then remove them before feeding it to TreeShrink or rooted pruning. We create two sets of data, one with 5% and the other with 10% of the sites changed, each consists of 20 replicates. The trees in this experiment are estimated by FastTree.

## Results

We start by comparing the three tests currently implemented in TreeShrink. We then compare the per-species test of TreeShrink with alternative methods.

### Comparing the three tests of TreeShrink

#### The impact of filtering on taxon occupancy

The three tests of TreeShrink (*α*=0.05) impact taxon occupancy differently, especially for outgroups. Outgroups naturally impact the tree diameter, but ideally, they should not be removed more often than other leaves. In all six datasets, the per-gene and all-gene tests tend to remove outgroups aggressively, while the per-species test removes all species, including outgroups, close to uniformly (Table 2 and Additional file 1: Figure S3).

**Table 2:** The impact of the three tests of TreeShrink on taxon occupancy

Dataset	Method	Portion of data removed(%)	Portion of outgroups removed(%)
Plants	Per-gene	3.3	29.9
	All-gene	2.5	12.8
	Per-species	4.9	5.1
Mammals	Per-gene	0.6	11.8
	All-gene	1.2	17.0
	Per-species	3.6	4.7
Cannon	Per-gene	1.4	6.2
	All-gene	1.3	4.7
	Per-species	3.5	5.0
Rouse	Per-gene	1.3	1.9
	All-gene	1.2	1.1
	Per-species	4.0	4.5
Insects	Per-gene	1.2	6.6
	All-gene	0.8	2.9
	Per-species	4.3	5.0
Frogs	Per-gene	1.3	26.7
	All-gene	0.8	15.9
	Per-species	2.7	4.5

The most severe case is chicken, the sole outgroup in the Mammalian dataset. Chicken is removed in 12% of the genes by the per-gene test (19 times more than the average) and in 17% by the all-gene test (13 times more than the average). Note that in this dataset, both per-gene and all-gene tests remove only around 1% of the data, so the frequent removal of the chicken corroborates our suspicion that TreeShrink used with per-gene or all-gene tests can remove outgroups often even if the outgroup sequence contains no errors. The per-species test, on the other hand, only removes chicken slightly more often than the average: it removes about 4% of the overall data and removes chicken in about 5% of the genes that have it (Figure S3b in Appendix B, Additional file 1). In addition to the outgroups, platypus is also removed often. Being basal to the other mammals, platypus is prone to the same issues as outgroups. However, there is also some evidence that platypus is often misplaced in many gene trees of this dataset [37]. Just as the chicken, platypus is removed significantly more often than other species: 5% in the per-gene test (7 times more often than the average) and 13% in the all-gene test (10 times more often than the average). Again, the per-species test removes platypus just slightly more often than the average: platypus is removed in about 5% of the genes while the average of all species is 4% (Figure S3 in Appendix B, Additional file 1).

#### The impact of filtering on gene tree discordance

**Fig. 4:**
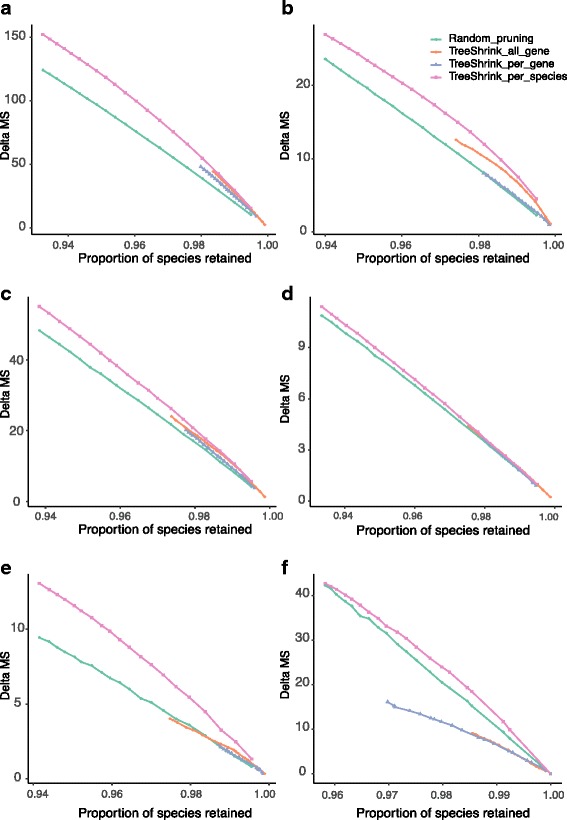
The impact of the three versions of TreeShrink on gene tree discordance on six datasets comparing to random pruning. MS distances are computed for all pairs of gene trees. The average reduction in the MS distance (*y-axis*) is shown versus the total proportion of the species retained in the gene trees after filtering (x-axis). A line is drawn between all points corresponding to different thresholds of the same method. **a** Insects, **b** Plants, **c** Metazoa - Canon, **d** Metazoa - Rouse, **e** Mammals, **f** Frogs

We now compare the three tests of TreeShrink in reducing gene tree discordance with minimal filtering. A method is preferred when it reduces the discordance more for a given level of filtering (i.e., higher lines in Fig. 4 are preferred). Except for the Frogs dataset, all the three tests of TreeShrink are on average better than the control random pruning. On the Frogs dataset, however, only the per-species test is better than the control. The failure of the other two tests could be because they remove outgroups often (see Table 2) and fail to remove the true outliers (perhaps because the true outliers are masked by the outgroups). Overall, the per-species test is consistently the most effective, followed by the all-gene test, and finally the per-gene test. Differences between the per-species test and the all-gene tests are substantial for plants, mammals, and frogs datasets, and less pronounced for others. Since the per-species test of TreeShrink is consistently the best here, we recommend using the per-species test for phylogenomic datasets which contain many genes.

### Comparing TreeShrink per-species with RogueNaRok and rooted pruning

We now compare TreeShrink per-species with alternative filtering methods.

#### The impact of filtering on taxon occupancy

**Fig. 5:**
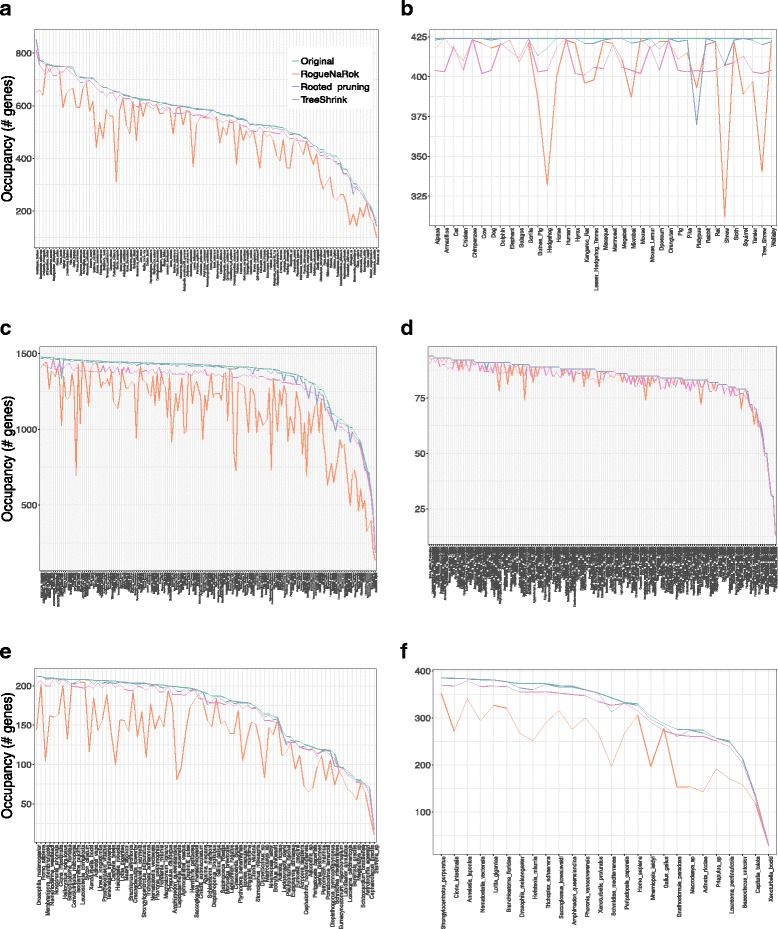
The impact of filtering on taxon occupancy for the six datasets. For each taxon (x-axis, ordered by occupancy), we show the number of genes that include it before and after filtering. **a** Plants, **b** Mammals, **c** Insects, **d** Frogs, **e** Metazoa - Cannon, **f** Metazoa-Rouse

Methods run in the default settings (*α*=0.05 for TreeShrink, 3 std for rooted pruning) impact occupancy differently. Overall, RogueNaRok reduces the occupancy more than the other methods (Fig. 5). Single species at the base of large clades seem especially prone to filtering by RogueNaRok. In contrast, TreeShrink and rooted pruning do not remove any specific taxon extensively.

On the plants dataset (Fig. 5a), RogueNaRok removes three species from at least half of the gene trees where they are present and removes 12 species from one-third of the genes. Examples include *Kochia scoparia* (removed in 343 out of 654 genes), *Acorus americanus* (251/693), and *Larrea tridentata* (221/590) genes. *Kochia scoparia* is on a long branch and sister to a group of 7 Eudicots, and *Acorus americanus* is basal to 10 Monocots [23]. Surprisingly, *Arabidopsis thaliana* is removed in 200 genes, even though it is a genome and is presumably less error-prone compared to most other transcriptomes species. Moreover, a focal point of this study is placing *Chara vulgaris* as basal to all land plants plus two algal groups (Zygnematophyceae, and Coleochaetales). RogueNaRok removes Chara from 160 genes out of 302 that include it; such aggressive filtering could limit the ability to answer this main biological question with confidence. In contrast, rooted pruning and TreeShrink remove 4 and 7% of the data, respectively. TreeShrink never removes any species in more than 6% of the genes and all species are removed in similar proportions.

On the insects dataset (Fig. 5c), RogueNaRok removes 17% of all the data and many removed species are basal to large diverse groups. For example, RogueNaRok removes *Conwentzia psociformis*, which is basal among 8 Neuropterida [31] from 684 out of 1412 genes that included it. *Zorotypus caudelli*, an enigmatic species placed as sister to a large clade in the ASTRAL species tree is also removed from 52% of the genes. Interestingly, RogueNaRok removes several outgroups, including *Speleonectes tulumensis* and *Cypridininae sp* frequently (56 and 57%). In contrast, rooted pruning and TreeShrink only remove a minimal amount of data (1 and 4%, respectively) and do not impact occupancy dramatically for any species.

Similar patterns are observed on Metazoa datasets (Fig. 5e, f). RogueNaRok removes more than 20% of the leaves overall, and many species are extensively removed from many genes. In the Cannon dataset, *Xenoturbella bocki* is removed in 93 out of the 208 genes that included it. Xenoturbella is the basal branch of the Xenacoelomorpha and in this study, is one of the most important species; removing it in 45% of genes would leave a long branch and could negatively impact the placement of Xenacoelomorpha. Rooted pruning and TreeShrink, again, remove a minimal portion of the data (2 and 4%, respectively) and no species is extensively removed.

The mammalian dataset is not extensively filtered by any method (Fig. 5b). Rooted pruning only removes about 1% of the data, while RogueNaRok and TreeShrink remove about 4%. RogueNaRok removes three species (shrew, tree shrew, and hedgehog) relatively often (i.e., > 80 genes). The shrew and the hedgehog are both basal branches to a larger clade of Laurasiatheria. The tree shrew has a very uncertain position in various species trees estimated on this dataset [6, 30, 36, 37]; RogueNaRok results indicate that its position is also unstable in gene trees. Platypus is also removed relatively often by rooted pruning (54 times), but somewhat less frequently by RogueNaRok (31 times) and TreeShrink (20 times). Several issues in the platypus sequences have been identified [37], and perhaps, its extensive filtering by rooted pruning is justified. Similar to the mammalian dataset, on the frogs dataset (Fig. 5d), all methods remove very little data (< 3% overall).

#### The impact of filtering on gene tree discordance

**Fig. 6:**
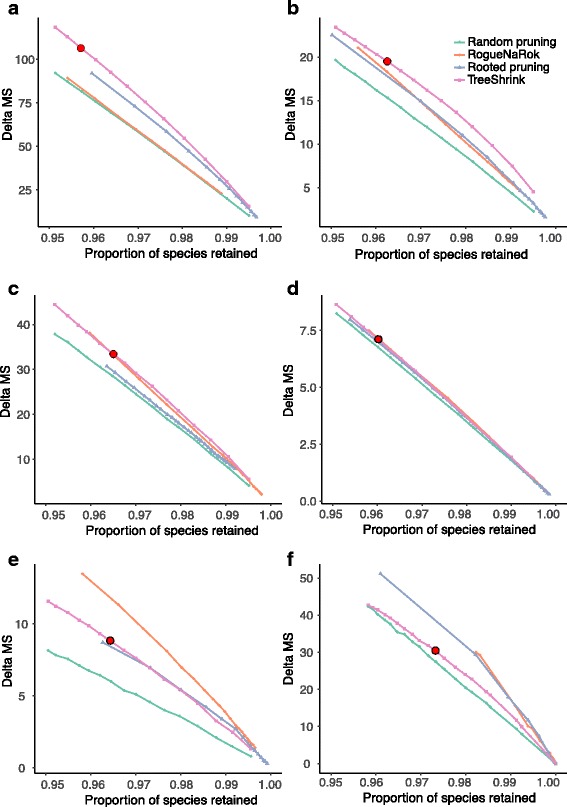
The impact of TreeShrink, RogueNaRok, and rooted pruning on gene tree discordance on six datasets comparing to random pruning. MS distances are computed for all pairs of gene trees. The average reduction in the MS distance (*y-axis*) is shown versus the total proportion of the species retained in the gene trees after filtering (x-axis). A line is drawn between all points corresponding to different thresholds of the same method. The points corresponding to the default setting of TreeShrink (*α*=0.05) are marked in red. **a** Insects, **b** Plants, **c** Metazoa - Cannon, **d** Metazoa - Rouse, **e** Mammals, **f** Frogs

Since extensive filtering is neither intended nor desired in this section, we focus on filtering thresholds that result in removing at most 5% of the data (see Figure S4 in Appendix B, Additional file 1, for the full data). On all six datasets, all the three filtering methods are on average better than the control random pruning. Comparing TreeShrink and the two alternatives, different patterns are observed on various datasets (Fig. 6).

On the two datasets with the largest numbers of genes, Plants and Insects, TreeShrink outperforms the other methods substantially (Fig. 6a, b). On the Insects dataset, RogueNaRok barely outperforms random pruning and TreeShrink is substantially better than rooted pruning. On the Plants dataset, rooted pruning and RogueNaRok are essentially tied and TreeShrink is consistently better than both. For example, TreeShrink with a 0.03 threshold removes 1476 species in total from all genes and reduces the average pairwise MS discordance by 15 units (as opposed to 11 for the control), whereas RogueNaRok and rooted pruning need to remove 1649 and 1740 species to achieve a reduction of up to 15 units in the MS discord.

On the Metazoa-Cannon dataset (Fig. 6c), TreeShrink and RogueNaRok both outperform rooted pruning, and TreeShrink has a small but consistent advantage over RogueNaRok. On the Metazoa-Rouse dataset, all methods are comparable and barely outperform random pruning (Fig. 6d).

On the Mammalian dataset (Fig. 6e), RogueNaRok is by far the best, followed by TreeShrink and rooted pruning, which have similar overall performance. On the Frogs dataset, which included only 95 genes, RogueNaRok and rooted pruning are tied and both substantially outperform TreeShrink (Fig. 6f).

Overall, TreeShrink is the best or tied with the best method in four datasets, and is outperformed in the other two. TreeShrink seems especially well suited for datasets with a large number of genes.

### The HIV dataset

#### Detecting non-subtype B sequences

**Fig. 7:**
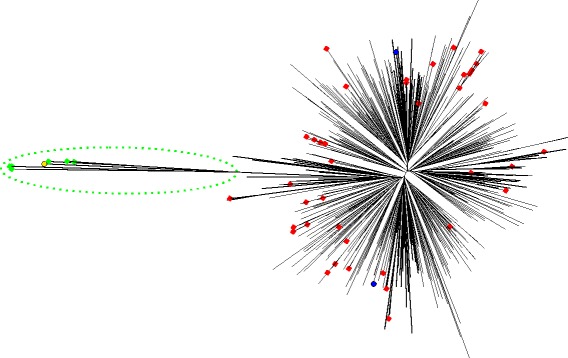
The HIV Tree. The subtype G sequence that could be detected by both TreeShrink and RogueNaRok is marked in yellow. The other non-subtype B sequences that could be detected by TreeShrink are marked in green. The subtype B species that were detected by RogueNaRok are marked in red. The two unassigned sequences are marked in blue

Using the RAxML tree of the 648 HIV *pol* sequences as input, TreeShrink correctly detects all seven non-subtype B sequences, including a single subtype CRF01 _AE sequence, two CRF02 _AG sequences, three subtype C sequences, and a subtype G sequence. The two unassigned sequences are not identified as outliers by TreeShrink (Fig. 7). Importantly, TreeShrink does not remove any subtype B sequences. In contrast, RogueNaRok identifies 41 rogue sequences in total, only one of which is non-subtype B (the subtype G sequence KJ723366). As we elaborate in the discussions, these differences are due to different objectives of the two methods. With midpoint rooting, rooted pruning detects three non-subtype B sequences (i.e., CRF01 _AE and two CRF02 _AG) as outliers but it misses the other 4 non-subtype B sequences and has two false positives.

#### Detecting simulated outliers

**Fig. 8:**
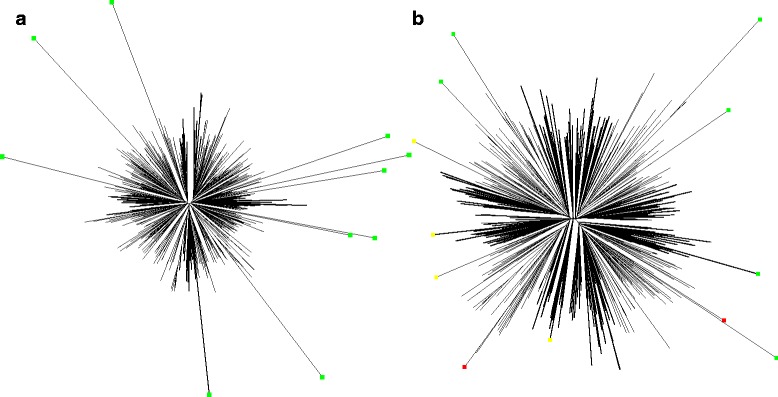
Examples of two HIV trees with 10 leaves of 10 and 5% changed in sequence. The true positives, false positives, and false negatives of TreeShrink detection (default settings) are marked in green, red, and yellow, respectively. **a** 10% error, **b** 5% error

Recall that for each simulated dataset, we have 20 replicates and each consists of 10 simulated outliers, for the total of 200 outliers to be detected. On the dataset with outliers at 10% changed in sequences, TreeShrink correctly detects 198/200 outliers and rooted pruning detects all 200/200 outliers; neither method has a false positive. On the dataset with outliers at 5% changed in sequences, TreeShrink correctly detects 106/200 outliers with 9 false positives while rooted pruning detects 131/200 outliers with 17 false positives. Overall, TreeShrink has higher precision and specificity but lower sensitivity comparing to rooted pruning (Table 3), indicating that TreeShrink is a more conservative approach. Figure 8 shows one example for each of the two simulation settings.

**Table 3:** Performance of TreeShrink in detecting simulated outliers

Dataset	Method	True positives	False positives	Precision	Recall (Sensitivity)	Specificity
5% changed	TreeShrink	106	9	92.2%	53.0%	98.6%
	Rooted pruning	131	17	88.5%	65.5%	97.3%
10% changed	TreeShrink	198	0	100%	99.00%	100%
	Rooted pruning	200	0	100%	100.00%	100%

Each of the two datasets consists of 20 replicates, each has 639 HIV-1 subtype B sequences and 10 simulated outliers, for the total of 12780 subtype B HIV sequences and 200 simulated outliers

## Discussions

It has been noted before that extreme long branches in a phylogeny can be erroneous. Gatesy and Springer used the presence of long branches in gene trees estimated in two mammalian datasets to argue against specific coalescent-based analyses (see Figs. 9 and 10 of their paper [7]). To eliminate problematic long branches, a typical approach is to root the tree and filter out leaves too distant from the root [23, 26]. TreeShrink can automatically filter out such outliers *without* rooting. In addition, TreeShrink is very scalable. It could finish processing the GreenGenes tree [44] with 203,452 leaves (*k*=2255) in 28 min and identified 39 species that could be filtered.

In this study, we observed that the per-species test of TreeShrink is consistently the best strategy, followed by the all-gene and the per-gene tests. However, it should be noted that the per-species test requires more data than the two alternative tests, and its data requirement has some practical implications. Because it relies on computing a distribution per species by aggregating data from all gene trees, the per-species test may degrade in performance when few genes are available. Consistent with this observation, we observed that the only dataset where the per-species test of TreeShrink was outperformed by rooted pruning was the Frogs dataset, which has fewer than a hundred gene trees (less than half of any other datasets). Similarly, the per-species test may not have enough information for species that have extremely low occupancy, to begin with. Therefore, we recommend caution in taking the suggestions of the per-species test for low-occupancy species.

We only examined effects of filtering leaves from existing trees without redoing alignments or gene trees after filtering. This was mostly due to our inability to replicate the exact analysis pipelines of every dataset we analyzed. When used on novel datasets, it is better to reestimate alignments and gene trees after the problematic sequences have been removed, because the problematic sequences could have negatively impacted gene alignments and gene trees of the remaining sequences.

Although we compared our method to RogueNaRok as an alternative to our approach, we point out that the two methods have different objectives and can complement each other. While RogueNaRok aims to remove *rogue species* based on topological stability, TreeShrink detects and removes *erroneous species* based on tree diameter. An analysis pipeline could use a combination of the two methods to find both erroneous sequences and difficult unstable tips of the phylogenetic tree. Our HIV dataset is a case in point. The differences between TreeShrink and RogueNaRok on this dataset can be mainly attributed to their different objectives. TreeShrink is specialized for detecting outlier species and is well-suited for specific applications such as screening of sub-types, finding contamination, or perhaps even finding paralogs. RogueNaRok, on the other hand, is designed to find species with unstable positions. Thus, our results should not discourage the use of RogueNaRok. Rather, the HIV example, and our results more broadly, are meant to clarify that shrinking the tree diameter can be an orthogonal approach to rogue taxon removal.

## Conclusions

In this paper, we introduced TreeShrink, a method to remove species that disproportionately impact a phylogenetic tree diameter *without* rooting. The tool is fully automatic and is publicly available. In our study, we showed that TreeShrink is highly accurate in screening subtypes of HIV, and is effective in reducing gene tree discordance in phylogenomic datasets. As a complement to the state-of-the-art rogue taxon removal tools, TreeShrink can be a new component to an analysis pipeline for screening sub-types, filtering contamination, and detecting paralogs.

## Additional file

Additional file 1 Supplementary material. Appendix A — Theorem proofs. Appendix B — Supplementary figures and tables. (PDF 399 kb)
