# Supplementary material for: TreeShrink: fast and accurate detection of outlier long branches in collections of phylogenetic trees
Source: BMC Genomics. 2018 May 8;19(Suppl 5):272. doi: 10.1186/s12864-018-4620-2 (PMC5998883; doi:10.1186/s12864-018-4620-2)
Supplement: Supplementary file 1 — Supplementary material. Appendix A — Theorem proofs. Appendix B — Supplementary figures and tables. (PDF 399 kb) [file 12864_2018_4620_MOESM1_ESM.pdf]

Supplementary Material

Appendix A: Theorem proofs

Proof of Proposition 1

We start with a Lemma:

Lemma S1 *If (a, b) is a diameter pair of t, then for any $c, d \in \mathcal{L} - \{a\}$,*

$$max(\delta(c, b), \delta(d, b)) \geq \delta(c, d) \ .$$

Proof Consider the quartet formed by the 4 leaves a, b, c, d in t.

Case 1: (Figure S1c) a and b are on the same side of the quartet:

$$\delta(a, b) \geq \delta(a, d) \implies \delta(m, b) \geq \delta(m, d) \implies \delta(c, m)+\delta(m, b) \geq \delta(c, m)+\delta(m, d) \implies \delta(c, b) \geq \delta(c, d)$$

Case 2: (Figure S1d) Without loss of generality, we assume $\delta(n, c) \geq \delta(n, d)$. We will prove that $\delta(b, c) \geq \delta(c, d)$.

We have:

$$\delta(a, b) \geq \delta(a, c) \implies \delta(b, m) \geq \delta(c, m)$$

$$\implies \delta(b, m) + \delta(n, c) \geq \delta(c, m) + \delta(n, c)$$

$$\implies \delta(b, m) + \delta(n, c) \geq 2\delta(n, c)$$

$$\implies \delta(b, c) = \delta(b, m) + \delta(n, c) + \delta(m, n) \geq \delta(n, c) + \delta(n, d) = \delta(c, d) \ .$$

□

We now provide the proof of Proposition 1.

Proof Consider an arbitrary leaf $b \in \mathcal{D}(t) - \{a\}$. We prove that $b \in \mathcal{D}(t\backslash_a)$.

Case 1: $(a, b) \notin \mathcal{P}(t)$. Because $b \in \mathcal{D}(t)$, there exists $c \in \mathcal{D}(t) - \{a\}$ such that (c, b) is a diameter pair of $t\backslash_a$. Therefore, $b \in \mathcal{D}(t\backslash_a)$.

Case 2: $(a, b) \in \mathcal{P}(t)$. Let (c, d) be a diameter-pair of $t\backslash_a$. According to Lemma S1, $max(\delta(c, b), \delta(d, b)) \geq \delta(c, d)$. Therefore, either (c, b) or (d, b) is a diameter-pair of $t\backslash_a$. Thus, $b \in \mathcal{D}(t\backslash_a)$. □

Proof of Theorem 1

Proof We need to prove that a $\mathcal{R}_k(t)$ is either a reasonable removing set or it is not an optimal removing set. We proceed by contradiction. Assume $\mathcal{R}_k(t)$ is optimal but not a reasonable removing set. Let $\mathcal{R}_m(t)$ be the largest reasonable removing set that is a subset of $\mathcal{R}_k(t)$ (note $0 \leq m \leq k$). If $m = k$, then $\mathcal{R}_k(t)$ is a reasonable set, contradicting the assumption. For $m < k$, consider the tree $t \upharpoonright_{\mathcal{R}_m(t)}$ and let a_m, b_m be its diameter pair. if $a_m \in \mathcal{R}_k(t)$ or $b_m \in \mathcal{R}_k(t)$, adding them to $\mathcal{R}_m(t)$

would generate a reasonable chain of size $m+1$, contradicting our assumption. If $a_m \notin \mathcal{R}_k(t)$ and $b_m \notin \mathcal{R}_k(t)$, all removals after m in $\mathcal{R}_k(t)$ fail to reduce the diameter, but removing either a_m or b_m would reduce the diameter. Thus, $\mathcal{R}_k(t)$ cannot be optimal, contradicting our assumption. □

Proof of Theorem 2

Proof To remove k leaves from a singly paired tree t that has (a, b) as a diameter pair, at least one of a or b has to be removed (or else the diameter never decreases). Thus, three types of reasonable chains exist: those that contain only a, those that contain only b, and those that contain both a and b. Note that after removing a, by Proposition 1, removing b is a reasonable removal (and vice versa), and thus, removing both a and b is always reasonable (in either order).

Case 1: $a \in \mathcal{R}_{k-2}, b \notin \mathcal{R}_{k-2}$: If a reasonable chain has a but not b, by Proposition 1, b is in the diameter set at each step of the chain. Since b by definition is never removed and recalling that the tree is singly paired, at each step, there is only one reasonable removal (whatever leaf is on-diameter in addition to b). Therefore, only one reasonable chain does not include b.

Case 2: $a \notin \mathcal{R}_{k-2}, b \in \mathcal{R}_{k-2}$: Similar to Case 1, one such chain exists.

Case 3: $a \in \mathcal{R}_{k-2}, b \in \mathcal{R}_{k-2}$: In this case, the reasonable removing chain must start with a, b or b, a. In either ordering, we are left with the same induced tree, and need to remove $k-2$ more leaves. Therefore, the set of all reasonable removing sets in this case is: $\{(\{a, b\} \cup R) \text{ for } R \text{ in } \mathcal{S}_{k-2}(t\restriction_{\mathcal{L}-\{a,b\}})\}$.

Combining the three cases together, we have:

$$|\mathcal{S}_k(t)| = |\mathcal{S}_{k-2}(t\restriction_{\mathcal{L}-\{a,b\}})| + 2$$

Let $s_k = |\mathcal{S}_k(t)|$. We have the following recursion:

$$s_k = \begin{cases} 1, & k = 0 \\ 2, & k = 1 \\ s_{k-2} + 2 & k \geq 2 \end{cases} \tag{S1}$$

Thus, $s_k = k + 1$.

□

Proof of Proposition 2

Proof Recall that the record of each internal node keeps track of the most distant leaves below two children of the node. When we remove a, only those nodes on the path from a to the root can have a change in their record. The first traversal of Algorithm 1 updates the records for those nodes, using simple recursive functions that can be computed in $O(1)$ per node.

According to Proposition 1, $b \in \mathcal{D}(t\backslash_a)$. Therefore, one of the longest paths in $t\backslash_a$ must include b; let c be the other leaf. The record of the LCA of c and b, after the update in the first round, will have the value of this longest value. Thus, by checking the updated record for all nodes in the path from b to the root we will find the maximum value. Moreover, when updating the records in the first traversal

from a to the root, we have already checked all the nodes from the $LCA(a,b)$ to the root. In the second traversal, we check the nodes from b to $LCA(a,b)$, completing the search. Each of the two traversals of Algorithm 1 visits at most h nodes and only need constant time operations in each visit. Therefore, the overall time complexity of Algorithm 1 is $O(h)$. □

Proof of Theorem 3

First, we prove the following lemmas:

Lemma S2 *All the longest paths in any tree have the same midpoint.*

Proof If t has only one diameter pair, then Lemma S2 is trivially correct.

If t has more than one diameter pair, let (a,b) and (c,d) be two distinct diameter pairs of t and let m be the midpoint of the path between a and b. We prove that m is also the midpoint of the path between c and d, that is m lies on that path between c and d and $\delta(m,c) = \delta(m,d)$. w.l.o.g, we suppose $\delta(m,c) \geq \delta(m,d)$.

- We prove that the path between c and d must pass m; that is, c and d belong to two different subsets in the partition defined by m on $\mathcal{L}$ (we call elements of the partition a "side"). We prove by contradiction, assuming c and d belong to the same side of m. Then $\delta(m,c) + \delta(m,d) > \delta(c,d)$. Also, either a or b must be on a different side from c and d to m (by definition, a and b cannot be on the same side to m). Suppose a is in a different side from c and d to m. Then: $\delta(a,b) \geq \delta(a,c) \implies \delta(a,m) + \delta(m,b) \geq \delta(a,m) + \delta(m,c) \implies \delta(m,b) \geq \delta(m,c) \implies \delta(m,a) \geq \delta(m,c)$. So we have,
  $\delta(a,c) = \delta(m,a) + \delta(m,c) \geq 2\delta(m,c) \geq \delta(m,c) + \delta(m,d) > \delta(c,d)$; this leads to a contradiction because (c,d) is a diameter pair.
- Prove that $mc = md$. Suppose $\delta(m,c) > \delta(m,d)$.
  We have : $2\delta(m,a) = 2\delta(m,b) = \delta(a,b) = \delta(c,d) \leq \delta(m,c) + \delta(m,d) < 2\delta(m,c)$. Therefore: $\delta(m,a) < \delta(m,c)$ and $\delta(m,b) < \delta(m,c)$.
  Case 1: c belongs to a different side of a to m. Then, $\delta(m,a) + \delta(m,c) = \delta(a,c) \implies \delta(m,a) + \delta(m,b) < \delta(a,c) \implies \delta(a,b) < \delta(a,c)$. This is a contradiction because (a,b) is a diameter pair of t.
  Case 2: c belongs to the same side of a to m. Then c belongs to a different side of b to m. Similar to case 1, in this case we can prove that $\delta(a,b) < \delta(b,c)$ which also leads to a contradiction.
  Thus, m is the midpoint of the path between c and d.

□

This lemma allows us to define some new concepts that are useful in the rest of the proof.

New definitions:

The single midpoint of any tree t partitions the diameter set into disjoint subsets; we call each of those subsets a *diameter group* of t (if the midpoint is in the middle of the branch, we have two diameter groups; a midpoint coinciding on an internal node would give three or more groups). We call any restriction of t with k leaves

removed a *k-optimal restricted tree* if no other restriction removing k leaves has a lower diameter. We call a tree t *k-shrinkable* if there exists a k-removing set that *strictly* reduces its diameter. We call any induced tree on t that has a smaller diameter than t a *shrunk tree* of t. Note that unless all but one of the diameter groups of a tree t are removed, the tree cannot shrink in diameter. When all but one of the diameter groups of a tree t is removed, we refer to the resulting tree as a *minimum shrunk tree* of t.

It is easy to see the following lemma.

Lemma S3 *For all a and b, $(a, b) \in \mathcal{P}(t)$ if and only if a and b belong to two distinct diameter groups.*

Now we prove a less obvious Lemma.

Lemma S4 *If tree t is k-shrinkable, any k-optimal restricted tree t^* can be induced from one of the minimum shrunk trees of t.*

Proof Because t is k-shrinkable, the diameter of t^* must be strictly smaller than the diameter of t. Suppose t^* is not an induced tree of any minimum shrink tree of t; then, t* has at least two leaves from two different diameter groups of t. Based on Lemma S3, t^* shares with t at least one diameter pair and therefore, has the same diameter as t, which is a contradiction. □

We now turn to the proof of Theorem 3. Recall:

Theorem 3. For any k, any arbitrary pair-restricted k-removing space includes at least one optimal k-removing set.

Proof If t is not k-shrinkable, any k-removing set is optimal and the result trivially follows. We now focus on a case where t is k-shrinkable.

Suppose t has m diameter groups:

$$D^1 = \{d_1^1, d_2^1, \ldots, d_{p_1}^1\}, D^2 = \{d_1^2, d_2^2, \ldots, d_{p_2}^2\}, \ldots, D^m = \{d_1^m, d_2^m, \ldots, d_{p_m}^m\} \; .$$

For $i = 1 \ldots m$, let $k^i = |\bigcup_{j \neq i} D_j|$ be the size of all groups except group i, and let t^i denote the minimum shrunk tree of t that excludes all groups $D^j, j \neq i$. Let $k^p = \max_i(k^i)$. For the tree t to be k-shrinkable, we need that $k^i \leq k$; thus, $k \geq k^p$.

To produce any minimum shrunk tree t^i with $k^i \leq k^p$, we can start from any removal (a, b) such that $a \in D^x$ and $b \in D^y$ (for $x \neq y$), and continue to produce t^i. To see this, note that if $x \neq y \neq i$, any chain that starts with either a or b and continues to select from any groups other than D^i will produce the minimum shrunk tree t^i after k^i removals. Now, w.l.o.g, consider $x = i$ and $y \neq i$. Then, consider the chain that starts by removing y and continues by removals from any group other than D^i. This chain will also produce t^i after k^i removals. In other words, each pair-restricted k-removing space of t can produce all the minimum shrunk trees t^i that have $k^i \leq k$.

Based on Lemma S4, when t is k-shrinkable, at least one of the minimum shrunk trees (say t_i^*) can induce any k-optimal restricted tree t^*. We also just proved that any pair-restricted space can produce *all* minimum shrunk trees. Therefore, any arbitrary pair-restricted removing space will include a chain that induces t_i^* from t and another chain that produces t^* starting from t_i^*. Thus, the union of the removing sets corresponding to these two chains will produce t^* and will be part of any arbitrary pair-restricted k-removing space. $\square$

Appendix B: Supplementary figures and tables

Table S1 Summary of the 9 outliers of the HIV dataset

Sequence ID	Subtype
KJ723095	CRF01_AE
KJ723070	CRF02_AG
KJ723094	CRF02_AG
KJ723062	C
KJ723387	C
KJ723455	C
KJ723366	G
KJ722966	unassigned (B or CRF01_AE)
KJ723048	unassigned (B or F1)

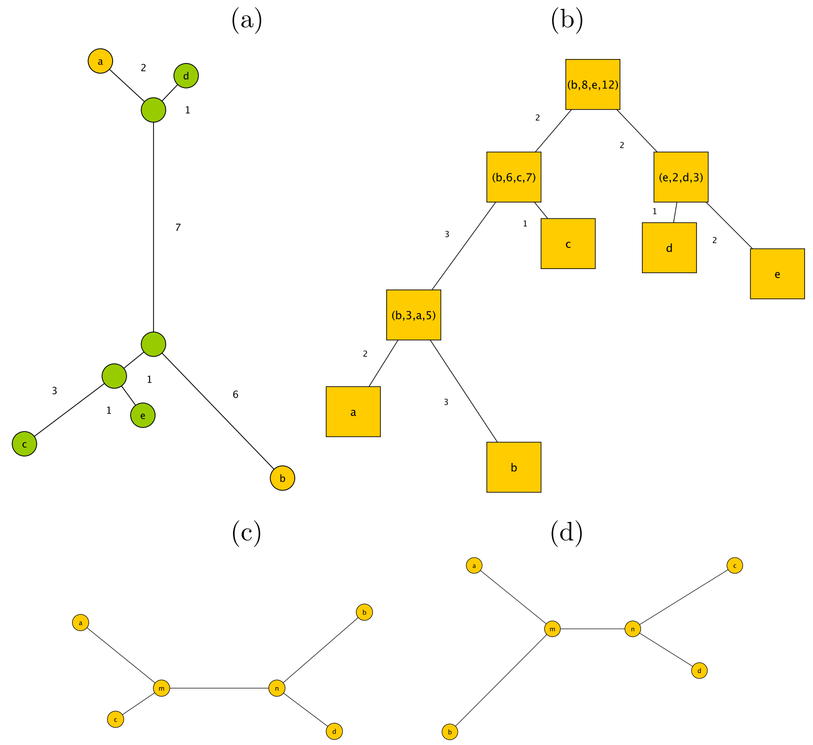

Figure S1 Demonstration of the k-shrink algorithm (*a*) An example where a greedy method does not work. This tree only has one diameter pair (a, b) (colored in yellow). If $k = 2$, the greedy method removes b and c and gives a restricted tree with diameter 11, while the optimal solution is to remove a and d, which gives the restricted tree with diameter 10. (*b*) The preprocessing step. In a post-order traversal, we store $rec(u)$ for each internal node u. The record has four values: leaf x under u that has the longest distance to u, the distance $\delta(u, x)$, the leaf y in one of the other sides of u to x that has the largest $\delta(x, y)$, and the distance. (c) and (d) Example quartet trees used in Proof of Lemma S1.

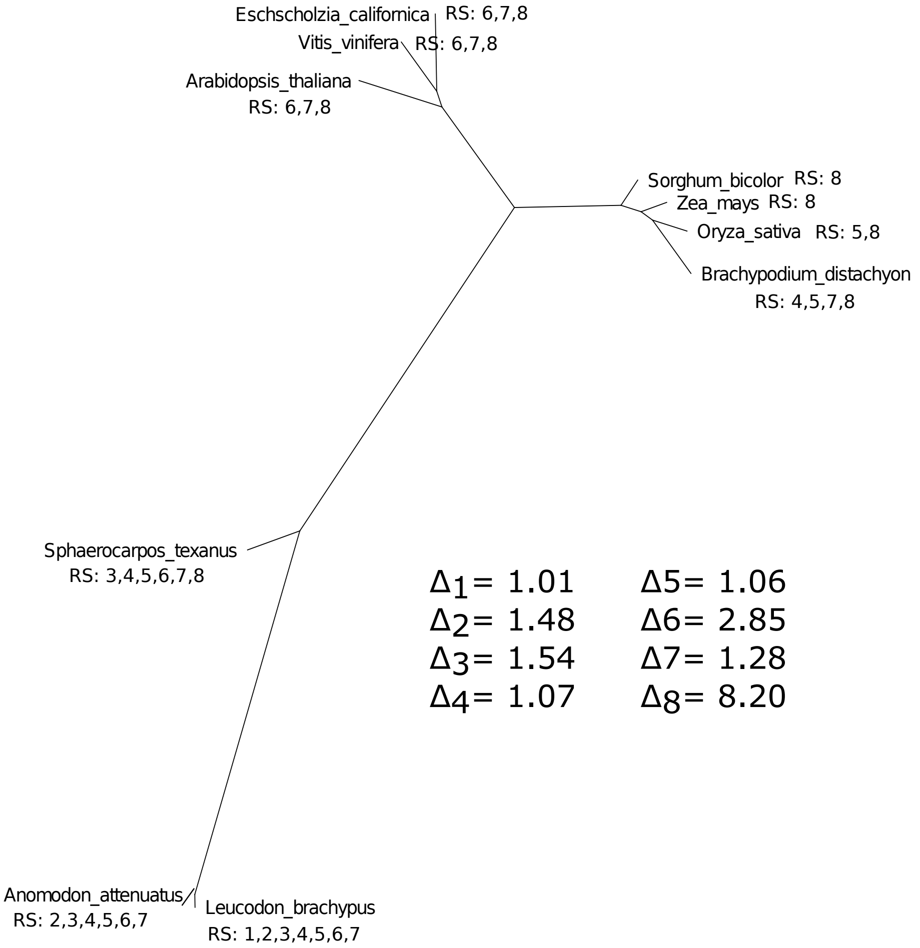

Figure S2 **An example of a very small tree from the Plants dataset.** Solving k-shrink with $k = 8$ gives the removing set of all but two species Anomodon attenuatus and Leucodon brachypus. Although this removing set is obviously wrong, it has a very high ratio value ($\nu_8 = 8.197$).

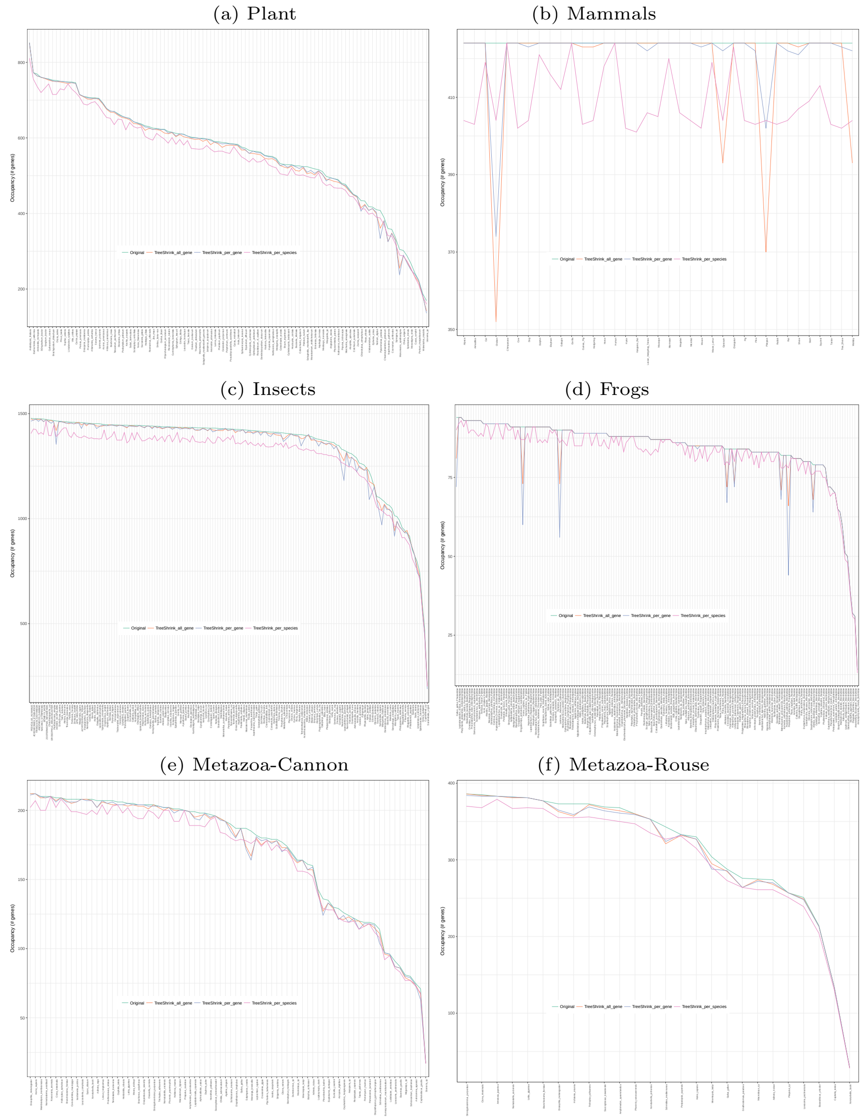

Figure S3 The impact of the 3 tests of TreeShrink on taxon occupancy for the six datasets. For each species (x-axis, ordered by occupancy), we show the number of genes that include it before and after filtering (default settings).

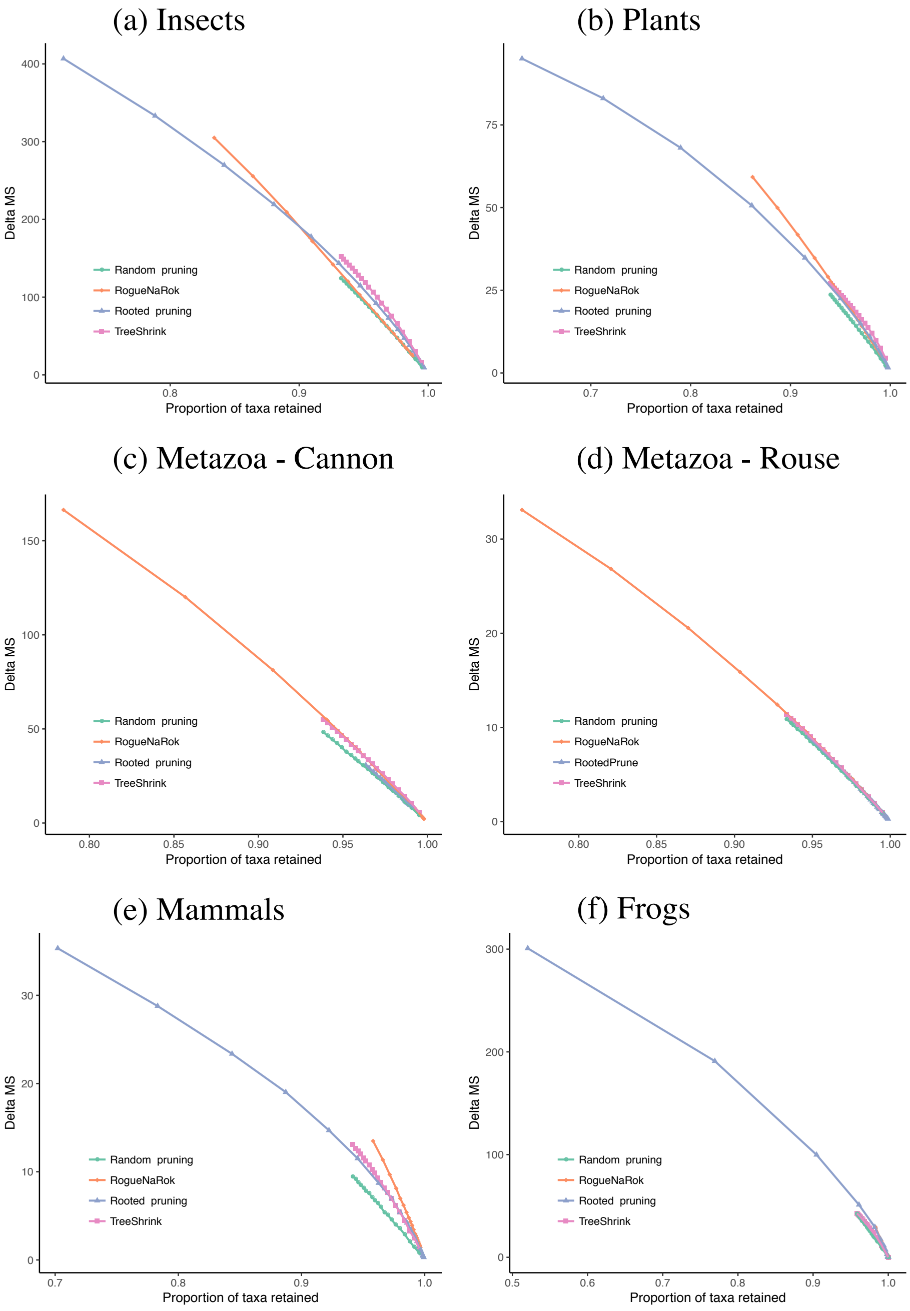

Figure S4 The impact of TreeShrink, RogueNaRok, and rooted pruning on gene tree discordance on six datasets comparing to random pruning. MS distances are computed for all pairs of gene trees. The average reduction in the MS distance (*y-axis*) is shown versus the total proportion of the species retained in the gene trees after filtering (x-axis). A line is drawn between all points corresponding to different thresholds of the same method.
